# Modulation of Free Amino Acid Profile in Healthy Humans Administered with Mastiha Terpenes. An Open-Label Trial

**DOI:** 10.3390/nu10060715

**Published:** 2018-06-03

**Authors:** Efstathia Papada, Ljilja Torović, Charalampia Amerikanou, Nikolaos Kalogeropoulos, Ilias Smyrnioudis, Andriana C. Kaliora

**Affiliations:** 1 Laboratory of Chemistry-Biochemistry and Physical Chemistry of Foods, Department of Dietetics and Nutritional Science, School of Health Science and Education, Harokopio University Athens, 17671 Athens, Greece; efpapada@gmail.com (E.P.); amerikanou@windowslive.com (C.A.); nickal@hua.gr (N.K.); 2 Department of Pharmacy, Faculty of Medicine, University of Novi Sad, 21000 Novi Sad, Serbia; ljilja.torovic@izjzv.org.rs; 3 Chios Mastic Gum Growers Association, 1 K. Monomachou St., 82100 Chios, Greece; ismyrnioudis@gummastic.gr

**Keywords:** plasma-free amino acids, terpenes, GC-MS metabolic analysis, uric acid

## Abstract

We aimed to explore whether plasma-free amino acids are modified in response to terpenes administration in healthy humans. In this open-label, single-arm acute trial, seventeen healthy male volunteers were administered with a naturally occurring product of known terpenes—namely mastiha—after overnight fasting. Blood samples were collected at different time points before and after ingestion. We aimed at identifying and quantifying 60 free amino acids in plasma applying Gas Chromatography-Mass Spectrometry. A total of 24 free amino acids were quantified. Branched-chain valine significantly decreased 4 h post-ingestion, whereas proline decreased at 6 h and ornithine at 2 h, compared to 0 h. These novel findings demonstrate that free amino acids levels are modulated in response to terpenes intake in healthy subjects.

## 1. Introduction

Amino acids (AAs) play key roles in pathways regulating intestinal health, participating in protein synthesis, gene expression, intracellular protein turnover, antioxidant/oxidant status and immunity [1,2]. Increased plasma-free AAs are correlated with insulin resistance in healthy non-obese individuals, independent of dietary protein intake [3]. Additionally, in healthy obese, hyperaminoacidemia is proposed as a sign of increased insulin resistance, and decreased plasma branched-chain amino acids (BCAAs) correlate with reduced insulin resistance after weight loss [4]. Regarding risk of developing future type 2 diabetes mellitus in healthy subjects, in the Framingham Offspring Study [5] and in a Finnish cohort [6], BCAAs were found predictive of diabetes. Plasma-free AAs have been linked with physiological conditions and variations in plasma are indicative of metabolic conditions [7].

Terpenes represent a heterogenous group of secondary metabolites of plants. They have been proposed as potential pharmacological therapeutic agents, as previous research has pointed towards their antioxidant, anti-inflammatory, anticancer, analgesic, immune modulating and wound healing properties [8]. Interestingly, a recent study has shown that the rich-in-terpenes methanolic extract of the fruit *Sechium edule* (cultivated from Mexico to South America and Antilles) inhibits specifically tumor cells and not normal cells [9]. Additionally, masticadienonic and 3α-hydroxymasticadienoic acids exhibit chemopreventive effects in experimental mice [10].

Overall, interventions with bioactive phytochemicals that alter plasma-free AA profile in humans are lacking. We have recently reported the absorption and bioavailability of a naturally occurring product—namely mastiha—that is a concentrated source of terpenes, mainly triterpenes, [11,12] and contains traces of simple phenols [13]. In particular, the major mastiha’s terpenes mastihadienonic and isomastihadienonic, but also other terpenes, such as moronic and oleanonic acids, are absorbed and bioavailable in plasma [14]. Concentrations of these main triterpenic compounds are known [11]. Herein, we aimed at understanding the in vivo effect of terpene intake in AA profiles in humans.

## 2. Materials and Methods

### 2.1. Study Design

**Figure 1:**
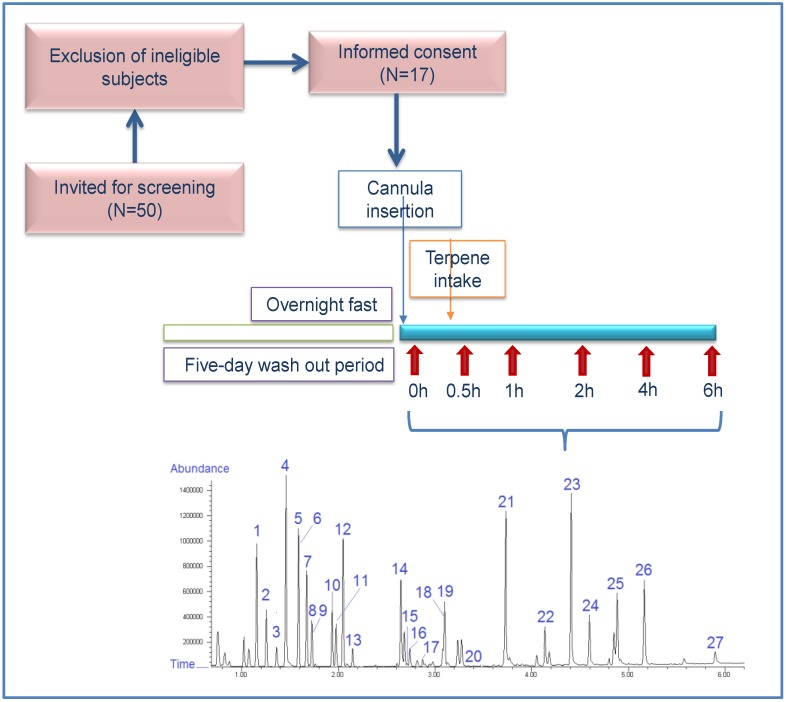
Study flowchart. (**1**) alanine; (**2**) glycine; (**3**) α-aminobutyric acid; (**4**) valine; (**5**) β-aminoisobutyric acid; (**6**) I.S. norvaline; (**7**) leucine; (**8**) allo-isoleucine; (**9**) isoleucine; (**10**) threonine; (**11**) serine; (**12**) proline; (**13**) asparagine; (**14**) thioproline; (**15**) aspartic acid; (**16**) methionine; (**17**) hydroxyproline; (**18**) glutamic acid; (**19**) phenylalanine; (**20**) α-aminoadipic acid; (**21**) glutamine; (**22**) ornithine; (**23**) lysine; (**24**) histidine; (**25**) tyrosine; (**26**) tryptophan; (**27**) cysteine.

The study was conducted in accordance with the Declaration of Helsinki of 1975 as revised in 2008. The protocol was approved by the Ethics Committee of Harokopio University of Athens (49/29–10–2015) and was registered as Mastiha BIO-GR in ClinicalTrials.gov (Identifier: NCT02847117). Free-living and healthy male adults were subjected to this acute and open-label intervention that has been previously described [14]. On the basis of an absorbance and bioavailability design of mastiha’s major terpenes, a 0.35 proportion of participants experiencing no increase in concentration of terpenes available in plasma—namely mastihadienonic acid and isomastihadienonic acid—at or before 6 h post-ingestion was assumed [14]. The pre-specified 95% confidence limits were between 26.3–44.7%. Thus, the estimated sample size needed was at least 14 subjects. This was the initial assumption to estimate the sample size required in our BIO-GR trial in order to assess the differences in plasma circulating terpenes after acute mastiha ingestion. Assessment of absorption and bioavailability of mastihadienonic (MNA), isomastihadienonic (IMNA), oleanonic (OA) and moronic (MO) acids in human plasma was the primary outcome of BIO-GR trial. Changes in plasma-free amino acids are a secondary outcome and this study herein is a sub-study of the BIO-GR trial. In brief, 17 volunteers eligible to participate gave their informed consent before inclusion in the study and signed an informed consent document after full explanation of the objectives, risks and benefits of the study. After enrollment, the volunteers underwent a medical and dietary assessment. Moreover, a blood sample was obtained for the evaluation of routine biochemical profile. Anthropometric data included body weight, height and body composition applying Bioelectric Impedance Analysis (Tanita, Arlington Heights, IL, USA, SA165A-0950U-3). After baseline clinical and anthropometric assessment, the volunteers were instructed to follow a low-phytochemical diet for five consecutive days, i.e., no fruits, vegetables, legumes, coffee, tea, alcoholic beverages, and chocolate, aiming at minimizing the concentration of circulating dietary phytochemicals in eligible participants. Compliance with the low-phytochemical diet was checked, applying LC-MS analysis after wash-out and overnight fasting at time point 0 h that was defined as the control experiment of this acute study design. Data from chromatographic analysis verified that targeted terpenes were undetected in plasma samples, thus subsequent increase in their levels would be attributed to mastiha administration solely [14]. In detail, on the day of the experiment and after overnight fasting, blood was collected at different time points using a plastic cannula adapted in an arm vein by specialized staff. As mentioned above, the first blood sample was collected at time point 0 h and then subsequent blood collection followed the intake of 10 g of mastiha (Figure 1). Blood samples were centrifuged at 3000 rpm for 10 min at 4 °C for plasma and serum isolation that were stored at –80 °C until further analysis.

### 2.2. Gas Chromatography-Mass Spectrometry Analysis

Gas Chromatography-Mass Spectrometry (GC-MS) was applied for the profiling of AAs in plasma samples in an Agilent (Waldbronn, Germany) series GC 6890 N gas chromatograph, coupled with an HP 5973 Mass Spectrometer detector (EI, 70 eV), split-splitless injector and an HP 7683 autosampler. The extraction and derivatization of AAs in plasma was carried out as described in the Phenomenex EZ:Faast™ Free (Physiological) Amino Acid Analysis by GC-MS manual. An aliquot (2 μL) of the derivatized samples was injected into the GC at a split ratio of 1:15. AA separation was achieved using a Phenomenex Zebron ZB-A AA analysis dedicated column (length = 10 m, internal diameter = 0.25 mm, film thickness = 25 μm). Carrier gas was high purity helium at constant flow of 1.1 mL/min. The injector and transfer line temperatures were 250 °C and 340 °C, respectively. Initial oven temperature was 110 °C, increased to 320 °C at 30 °C/min and held at 320 °C for 3 min. A selective ion monitoring (SIM) GC/MS method was applied for the detection of 26 AAs, based on the ±0.05 retention time (Rt) presence of a target and a qualifier ion, with the exception of β-aminoisobutyric acid with more than one qualifier ions. The retention times, target and qualifier ions of the AAs are shown in Table S1 (Supplementary Material). Quantification was carried out employing norvaline as internal standard and constructing reference curves for every AA by means of standard solutions.

### 2.3. Quantification of Uric Acid in Serum

The uric acid assay is based on the oxidation of uric acid (UA) in presence of enzyme uricase, which results in the production of H_2_O_2_. The reaction of H_2_O_2_ with a phenolic derivative and 4-aminophenazone is catalyzed by the enzyme peroxidase and produces a red-colored product. UA was quantified in serum (Biosis, Athens, Greece). In detail, blank, standard and serum samples were mixed with a working solution consisting of uricase, aminophenazone, a phenolic derivative and peroxidase. Next, the mixture was incubated at 37 °C for 5 min. After addition of distilled water, the absorbance was measured at 510 nm in a microplate spectrophotometer (Biotek, PowerWave XS2, Winooski, VT, USA) and was proportional to the concentration of UA in the sample.

### 2.4. Total Serum Oxidisability Assay

Serum samples were oxidized with copper sulfate as described previously [14]. The kinetics of oxidation were analyzed in terms of the lag time preceding oxidation and expressed in seconds (tLAG).

### 2.5. Statistical Analysis

Statistical analysis was conducted with the SPSS software (SPSS for Windows, version 20.0, SPSS Inc., Chicago, IL, USA). A repeated measures ANOVA was performed to detect differences in AA and UA levels. Post hoc testing was applied to perform specific comparisons to discover the origin of the differences. Pearson correlation coefficients were used to evaluate any relationship between AA concentrations and oxidant/antioxidant status in blood. Level of statistical significance was set at *p* < 0.05.

## 3. Results

Both wash-out and intervention periods were successfully completed by all the 17 participants, whose anthropometrics and biochemical profile were within the normal range (Table 1). None of the subjects consumed any vitamin or medication and none of them had a history of alcohol or drug abuse.

A total of 26 plasma-free AAs were detected and 24 of them were quantified. Table 2 presents plasma concentrations of free AAs at different time points.

Overall, analysis with repeated measures ANOVA showed significant differences in plasma concentrations of valine, proline and ornithine. Post hoc analysis revealed that the BCAA valine was significantly lower at T_4_ compared with baseline. Proline concentration decreased significantly at T_6_ compared with baseline and T_2_, whereas it was significantly lower at T_4_ compared with T_1_. Ornithine levels were significantly decreased at T_2_ compared with baseline.

UA concentrations were within the reference ranges at different time points. However, no significant changes were observed between different time points (Table 3).

Levels of free AAs in plasma were correlated with oxidant/antioxidant status in blood (Table S2, supplementary material). Alpha-aminobutyric levels were positively and moderately correlated with UA at T_0_ and T_4_. Additionally, leucine and serine concentrations were moderately associated with UA at baseline. Furthermore, there was a strong positive correlation between cysteine and UA on T_2_.

## 4. Discussion

In this sub-study of the Mastiha BIO-GR trial, we have used targeted GC-MS plasma-free AA analysis to identify alterations in AAs in healthy non-obese subjects in response to terpenes.

Interestingly, we detected some significant changes in valine, proline and ornithine. Valine, a BCAA with direct effects on glutamine and arginine synthesis and on the balance of BCAAs [15], was significantly decreased at T_4_ after mastiha administration. BCAAs, including valine, have been associated with metabolic dysfunctions [5,16], Therefore, the observed decrease in valine might be of importance. In addition, proline, an AA with direct and indirect effects on collagen synthesis, cellular redox state, stress response and immunity [15], was significantly decreased at the time point 6 h. Upregulation of proline biosynthesis is an oxidative stress response in mammalian cells [17], and so proline is increased in subjects with metabolic dysfunctions [18]. In this acute trial design, the decrease in proline levels is indicative of the regulation in proline metabolism due to increase in serum antioxidant potential 6 h post-ingestion [14]. Furthermore, ornithine was decreased at time point 2 h compared with baseline. Ornithine participates in ammonia detoxification as an intermediate of the urea cycle and contributes to mitochondrial integrity [15]. Recently, an inverse association of plasma ornithine with low-grade chronic inflammation in healthy humans was reported [19]. Herein, the change reported in plasma ornithine at T_2_ post-ingestion coincides with a peak in plasma concentration of MNA [14].

UA, which is the end product of purine metabolism, is a potent antioxidant acting as free radical scavenger and a chelator of transition metals. Nevertheless, it is also considered as a pro-oxidant since reactive oxygen species (ROS) are byproducts of its synthesis, and it seems to stimulate synthesis of proinflammatory molecules [20]. Although, we did not detect any significant changes in UA levels, α-aminobutyric acid, leucine and serine were positively associated with UA at baseline. Additionally, α-aminobutyric acid correlated with UA at T_4_ and cysteine was strongly associated with UA at T_2_. This relationship is supported by a recent study in Japanese subjects with or without lifestyle-risk factors for future cardiovascular disease exploring the association between plasma-free AAs and high levels of UA. Apart from significant correlations of plasma-free AAs with UA, significant inverse associations with high UA levels were observed for arginine, asparagine and threonine in healthy subjects as well, indicating the possibility of interplay between plasma-free AAs and UA [21]. However, hereby, whether the changes in AAs have a direct relationship with antioxidant status or whether the relationship between plasma-free AAs and UA is causal remain unanswered.

We have previously published an increase in lag time in serum lipoprotein oxidation assay following administration with mastiha terpenes [14]. Herein, the AAs found significantly modulated after acute administration—valine at T_4_, proline at T_6_ and ornithine at T_2_—were not correlated with increase in lag time in serum lipoprotein assay or with UA or with terpenes quantified in plasma [14] at the respective time points. However, some significant correlations between the identified plasma-free AAs and lag time or UA or plasma terpenes were observed at different time points and are presented in supplementary material (Table S2).

As regards oxidative stress, related biomarkers are presented in literature with several controversies. Uric acid itself is characterized as a “controversial” marker since its antioxidant effects are manifested only in the hydrophilic environment of biological fluids, such as serum or plasma, acting as a powerful scavenger of carbon-centered and peroxyl radicals, while it loses its ability to scavenge lipophilic radicals or break the radical chain propagation within lipid membranes. Serum lipid oxidizability assay is an ex vivo challenge model that is dependent on the circulating bioactive compounds and represents a biomarker of a highly relevant aspect of human health as oxidative stress. On the other hand, while glutathione is an important antioxidant, its measurement in our protocol of acute administration and measurement of antioxidant status post-ingestion would not be applicable, due to its high turnover time in plasma [22].

Data on the effect of terpenes in the metabolic profile of humans are limited. To the best of our knowledge, limonene—a lipophilic monoterpene—is the only terpene examined as regards metabolic changes in humans. The study of Miller and co-workers has shown that limonene supplementation regulates a series of plasma metabolites [23]. In our study terpene administration was found to modulate the AA profile in healthy humans. In the study of Schmedes et al. [24] a significantly reduced level of valine and isoleucine was observed after a lean-seafood intake. The authors hypothesized that this effect was rather due to direct endogenous effects on the host’s own metabolic pathways rather than to the contained AAs in diets, as the differences in AAs between control and intervention diets were insignificant [24]. Herein, administration of mastiha terpenes, namely MNA and IMNA, may have resulted in modulation of plasma-free AAs due to the formation of hydrogen bonds with AAs. As such, hydrogen bonds were detected between MNA and IMNA with AAs in the study of Vuorinen et al. [25].

Even if our study has some limitations, such as the absence of the full metabolic profile identification, these limitations are counterbalanced by several strengths. The wash-out period of phytochemicals and the overnight fasting before the acute experiment contributed to a clearer pattern of the metabolic response of AAs to terpenes, since dietary AAs were absent. In addition, our homogenous sample of healthy and under-no-medication young men (20–40 years old) of the same ethnicity and within the normal body mass index range is an important strength, since previous (or literature) data show that plasma-free AAs profile of adults differ by gender, age, body mass index and ethnicity.

## 5. Conclusions

This study showed that plasma-free AAs are modulated in response to terpenes in healthy non-obese adult males. Further research is needed to clarify whether modulation of AAs by phytochemical compounds may be of physiological relevance.

## Figures and Tables

**Table 1:** Baseline characteristics.

Anthropometrics	
BMI (kg/m^2^)	24.8 ± 0.7
Body fat (%)	17.7 ± 1.5
Total Body Water (%)	57.8 ± 1.2
Muscle mass (kg)	61.5 ± 1.0
Bone mass (kg)	3.2 ± 0.1
Biochemical profile	
Glucose (mg/dL)	94.4 ± 2.9
Urea (mg/dL)	36.4 ± 1.8
Creatinine (mg/dL)	1.0 ± 0.0
Total Cholesterol (mg/dl)	184.8 ± 9.4
Triglycerides (mg/dL)	93.2 ± 14.6
High-Density Lipoprotein (mg/dL)	52.3 ± 3.2
Low-Density Lipoprotein (mg/dL)	113.9 ± 8.0
Total Cholesterol/High-Density Lipoprotein ratio	3.7 ± 0.3

Data are presented as Mean ± Standard Error of Mean.

**Table 2:** Plasma concentrations (nmol/mL) of free amino acids at different time points in healthy men.

Amino Acid	C_0_	C_1/2_	C_1_	C_2_	C_4_	C_6_	P
Alanine	283.5 ± 79.4	271.1 ± 83.6	266.9 ± 75.8	271.2 ± 62.0	253.4 ± 64.3	248.4 ± 69.6	NS
Glycine	205.9 ± 24.0	206.6 ± 26.9	205.0 ± 29.3	212.6 ± 30.1	213.7 ± 34.1	198.2 ± 56.0	NS
α-Aminobutyric acid	25.2 ± 7.7	25.4 ± 7.6	25.9 ± 8.1	26.7 ± 8.0	25.6 ± 8.2	26.4 ± 8.0	NS
Valine	409.3 ± 65.7 ^1^	389.6 ± 57.2	379.5 ± 59.1	380.9 ± 42.9	354.8 ± 55.1 ^1^	344.3 ± 55.8	^1^*p* = 0.014
β-Aminobutyric acid	99.46 ± 3.2	98.9 ± 3.1	98.4 ± 2.7	97.7 ± 2.2	98.8 ± 2.5	99.0 ± 3.6	NS
Leucine	148.7 ± 26.9	140.8 ± 17.9	135.0 ± 15.3	136.6 ± 12.7	140.6 ± 13.4	141.9 ± 16.8	NS
Allo-isoleucine	63.6 ± 15.9	59.1 ± 11.6	55.3 ± 8.9	56.0 ± 7.0	55.2 ± 8.4	53.9 ± 9.7	NS
Isoleucine	74.2 ± 18.2	69.0 ± 13.3	64.4 ± 10.4	65.6 ± 8.4	64.6 ± 9.6	63.2 ± 11.1	NS
Threonine	124.4 ± 24.1	130.4 ± 26.7	126.9 ± 34.3	122.3 ± 30.4	124.8 ± 20.6	117.0 ± 25.5	NS
Serine	111.8 ± 20.8	113.7 ± 22.0	115.8 ± 26.2	110.1 ± 17.4	112.6 ± 18.4	106.9 ± 33.5	NS
Proline	238.9 ± 115.6 ^2^	238.1 ± 102.8	233.4 ± 113.4 ^3^	229.3 ± 99.1 ^4^	218.0 ± 109.5 ^3^	194.9 ± 102.7 ^2,4^	^2^*p* = 0.0283; ^3^ *p* = 0.036; ^4^ *p* = 0.040
Asparagine	47.7 ± 12.4	48.8 ± 12.4	48.2 ± 11.8	47.6 ± 10.4	50.2 ± 12.1	48.0 ± 10.9	NS
Aspartic acid	14.3 ± 7.4	13.7 ± 6.4	13.4 ± 7.5	14.3 ± 8.5	14.4 ± 9.2	14.3 ± 6.5	NS
Methionine	23.3 ± 7.6	23.7 ± 6.1	22.6 ± 5.2	22.7 ± 4.3	23.9 ± 4.7	21.7 ± 5.4	NS
Hydroxyproline	16.7 ± 3.9	17.5 ± 4.1	17.2 ± 4.6	16.5 ± 3.3	16.2 ± 3.2	15.5 ± 2.6	NS
Glutamic acid	26.9 ± 21.6	23.3 ± 15.6	23.1 ± 21.9	12.2 ± 9.1	13.3 ± 10.9	9.1 ± 6.8	NS
Phenylalanine	66.2 ± 11.7	65.4 ± 8.4	62.4 ± 10.6	62.0 ± 7.5	67.2 ± 14.7	63.4 ± 8.1	NS
Glutamine	352.8±96.0	351.5 ± 81.2	356.1 ± 86.1	373.0 ± 118.6	326.4 ± 94.9	334.0 ± 114.2	NS
Ornithine	54.2 ± 12.0 ^5^	50.9 ± 10.4	49.8 ± 11.1	45.8 ± 9.6 ^5^	47.9 ± 10.9	47.1 ± 8.8	^5^*p* = 0.043
Lysine	169.6 ± 48.5	164.6 ± 42.0	166.3 ± 52.8	160.6 ± 57.2	173.9 ± 44.4	172.7 ± 40.0	NS
Histidine	77.9 ± 18.1	76.6 ± 21.9	80.3 ± 22.2	77.6 ± 26.2	78.8 ± 22.3	76.1 ± 18.9	NS
Tyrosine	61.5 ± 21.7	60.7 ± 18.3	55.5 ± 16.7	52.2 ± 14.1	52.4 ± 12.2	48.2 ± 9.7	NS
Tryptophane	67.6 ± 13.5	67.3 ± 11.1	64.2 ± 15.1	60.9 ± 12.2	61.4 ± 12.1	59.5 ± 11.4	NS
Cysteine	30.0 ± 3.4	31.1 ± 2.8	31.7 ± 2.9	32.1 ± 5.6	30.1 ± 1.6	30.4 ± 3.4	NS

Values are mean ± standard deviation. Values sharing the same number as superscript differ significantly. Non-significant (NS).

**Table 3:** Serum concentrations of uric acid at different time points in healthy men.

Time Points (h)	C (mg/dL)
T_0_	3.6 ± 0.9
T_1/2_	3.4 ± 0.8
T_1_	3.7 ± 1.1
T_2_	4.3 ± 2.1
T_4_	3.6 ± 0.7
T_6_	3.5 ± 0.8
T_24_	3.5 ± 0.7

Values are mean ± standard deviation.

## Supplementary Materials

The following are available online at http://www.mdpi.com/2072-6643/10/6/715/s1, Table S1: Retention times, target and qualifier ions of the amino acids and internal standard. Table S2: Pearson coefficients for amino acids correlations with uric acid, serum antioxidant capacity and terpene levels at different time points.
