# Supplementary material for: Modulation of Free Amino Acid Profile in Healthy Humans Administered with Mastiha Terpenes. An Open-Label Trial
Source: Nutrients. 2018 Jun 3;10(6):715. doi: 10.3390/nu10060715 (PMC6024789; doi:10.3390/nu10060715)
Supplement: Supplementary file 1 [file nutrients-10-00715-s001.pdf]

Table S1. Retention times, target and qualifier ions of the amino acids and internal standard.

**Amino acid**	**Rt (min)**	**Target Ion (m/z)**	**Qualifier Ions (m/z)**
**Alanine**	1.161	130	88
**Glycine**	1.261	116	102
**α-Aminobutyric acid**	1.366	144	102
**Valine**	1.462	158	116
**β-Aminoisobutyric acid**	1.534	116	143, 172
**Norvaline (Internal Standard)**	1.590	158	72
**Leucine**	1.675	172	86
**Allo-isoleucine**	1.703	172	130
**Isoleucine**	1.731	172	130
**Threonine**	1.943	160	101
**Serine**	1.980	156	203
**Proline**	2.051	156	243
**Asparagine**	2.150	155	69
**Thioproline**	2.719	174	147
**Aspartic acid**	2.723	216	130
**Methionine**	2.742	203	277
**Hydroxyproline**	2.879	172	130
**Glutamic acid**	3.081	230	170
**Phenylalanine**	3.100	206	190
**α-Aminoadipic acid**	3.396	244	98
**Glutamine**	3.720	187	84
**Ornithine**	4.134	156	70
**Lysine**	4.407	170	128
**Histidine**	4.585	282	168
**Tyrosine**	4.877	206	107
**Tryptophan**	5.959	130	130
**Cysteine**	5.897	248	216

Table S2. Pearson coefficients for amino acids correlations with uric acid, serum antioxidant capacity and terpene levels on different time points.

Amino acid	Serum uric acid	Serum antioxidant capacity [14]	Plasma MNA [14]	Plasma IMNA [14]	Plasma OLEA [14]	Plasma MA [14]
Alanine			R=0.484(T_6)*	R=0.540 (T_6)	R=0.491 (T_4)*	
α-Aminobutyric acid	R=0.522 (T_0)*<br>R=0.524 (T_4)*					
Valine		R=0.604 (T_2)*				
β-Aminobutyric acid					R=0.574 ($T_{1/2}$)*	R=0.575 ($T_{1/2}$)*
Leucine	R=0.520 (T_0)*	R=0.555 (T_4)*	R=0.532 (T_1)*		R=0.490 (T_1)*	
Allo-isoleucine			R=0.642 (T_1)*	R=0.522 (T_1)*	R=0.656 (T_1)**	R=0.491 (T_1)*<br>R=0.514 (T_2)*
Isoleucine			R=0.646 (T_1)**	R=0.523 (T_1)*	R=0.654 (T_1)**	R=0.494 (T_1)*<br>R=0.501 (T_2)*
Threonine		R=-0.717 (T_6)**				
Serine	R=0.589 (T_0)*					
Proline					R=0.705 ($T_{1/2}$)**	R=0.647 ($T_{1/2}$)**
Aspartic acid		R=0.542 (T_0)*<br>R=0.540 ($T_{1/2}$)*				
Methionine		R=-0.563 (T_2)*		R=0.561 (T_6)*	R=0.537 ($T_{1/2}$)*	
Phenylalanine		R=-0.553 (T_2)*<br>R=-0.613 (T_6)**				
Glutamine					R=0.516 (T_4)*	R=0.565 (T_4)*
Ornithine		R=-0.545 (T_6)*		R=0.699 ($T_{1/2}$)**	R=0.641 ($T_{1/2}$)*	R=0.726 ($T_{1/2}$)**
Lysine		R=-0.718 (T_6)**				R=0.531 ($T_{1/2}$)*
Histidine		R=-0.613 (T_6)*	R=-0.504 (T_1)*<br>R=-0.574 (T_2)*	R=-0.576 (T_2)*	R=-0.519 (T_2)*	R=-0.548 (T_2)*
Tyrosine		R=-0.703 (T_6)**				
Tryptophane		R=-0.496 (T_2)*<br>R=-0.633 (T_6)**				
Cysteine	R=0.736 (T_2)**	R=-0.629 (T_2)*	R=-0.607 (T_2)*			

MNA: mastihadienonic acid; IMNA: isomastihadienonic acid; OLE: oleanonic acid; MA: moronic acid; *$p<0.05$; **$p<0.01$.
